# Serum folate, homocysteine and colorectal cancer risk in women: a nested case–control study

**DOI:** 10.1038/sj.bjc.6690305

**Published:** 1999-04

**Authors:** I Kato, A M Dnistrian, M Schwartz, P Toniolo, K Koenig, R E Shore, A Akhmedkhanov, A Zeleniuch-Jacquotte, E Riboli

**Affiliations:** Nelson Institute of Environmental Medicine and Kaplan Comprehensive Cancer Center, New York University School of Medicine, 341 East 25th Street, New York, NY, 10010, USA; Department of Clinical Laboratories, Memorial Sloan Kettering Cancer Center, 1275 York Avenue, New York, NY, 10021, USA; Unit of Nutrition and Cancer, International Agency for Research on Cancer, 150 Cours Albert-Thomas, Lyon, 69372, France

**Keywords:** colorectal cancer, folate, homocysteine, cohort study women

## Abstract

Accumulating evidence suggests that folate, which is plentiful in vegetables and fruits, may be protective against colorectal cancer. The authors have studied the relationship of baseline levels of serum folate and homocysteine to the subsequent risk of colorectal cancer in a nested case–control study including 105 cases and 523 matched controls from the New York University Women's Health Study cohort. In univariate analyses, the cases had lower serum folate and higher serum homocysteine levels than controls. The difference was more significant for folate (*P* < 0.001) than for homocysteine (*P* = 0.04). After ad'justing for potential confounders, the risk of colorectal cancer in the subjects in the highest quartile of serum folate was half that of those in the lowest quartile (odds ratio, OR = 0.52, 95% confidence interval, CI = 0.27–0.97, *P*-value for trend = 0.04). The OR for the highest quartile of homocysteine, relative to the lowest quartile, was 1.72 (95% CI = 0.83–3.65, *P*-value for trend = 0.09). In addition, the risk of colorectal cancer was almost twice as high in subjects with below-median serum folate and above-median total alcohol intake compared with those with above-median serum folate and below-median alcohol consumption (OR = 1.99, 95% CI = 0.92–4.29). The potentially protective effects of folate need to be confirmed in clinical trials. © 1999 Cancer Research Campaign


*British Journal of Cancer* (1999) **79**(11/12), 1917–1921
© 1999 Cancer Research Campaign
Article no. bjoc.1998.0305

### Serum folate, homocysteine and colorectal cancer risk in women: a nested case–control study

**I Kato[1], AM Dnistrian[2], M Schwartz[2], P Toniolo[1], K Koenig[1], RE Shore[1], A Akhmedkhanov[1], A Zeleniuch-Jacquotte[1] and E Riboli[3]**

[1]Nelson Institute of Environmental Medicine and Kaplan Comprehensive Cancer Center, New York University School of Medicine, 341 East 25th Street, New York, NY 10010, USA; [2]Department of Clinical Laboratories, Memorial Sloan Kettering Cancer Center, 1275 York Avenue, New York, NY 10021, USA; and [3]Unit of Nutrition and Cancer, International Agency for Research on Cancer, 150 Cours Albert-Thomas 69372, Lyon, France

**Summary** Accumulating evidence suggests that folate, which is plentiful in vegetables and fruits, may be protective against colorectal cancer. The authors have studied the relationship of baseline levels of serum folate and homocysteine to the subsequent risk of colorectal cancer in a nested case–control study including 105 cases and 523 matched controls from the New York University Women's Health Study cohort. In univariate analyses, the cases had lower serum folate and higher serum homocysteine levels than controls. The difference was more significant for folate ($P < 0.001$) than for homocysteine ($P = 0.04$). After adjusting for potential confounders, the risk of colorectal cancer in the subjects in the highest quartile of serum folate was half that of those in the lowest quartile (odds ratio, OR = 0.52, 95% confidence interval, CI = 0.27–0.97, *P*-value for trend = 0.04). The OR for the highest quartile of homocysteine, relative to the lowest quartile, was 1.72 (95% CI = 0.83–3.65, *P*-value for trend = 0.09). In addition, the risk of colorectal cancer was almost twice as high in subjects with below-median serum folate and above-median total alcohol intake compared with those with above-median serum folate and below-median alcohol consumption (OR = 1.99, 95% CI = 0.92–4.29). The potentially protective effects of folate need to be confirmed in clinical trials.

**Keywords:** colorectal cancer; folate; homocysteine; cohort study women

Colorectal cancer is the third commonest cancer in both women and men in developed countries. More than 130 000 new cases are expected to be diagnosed every year in the US (American Cancer Society, 1997). Although a genetic predisposition to the disease is recognized, ample differences in cancer incidence between countries, over time and among migrants have been interpreted as suggestive of a prominent role of environmental exposures, especially diet (Doll and Peto, 1981). A diet high in animal fat or red meat has been linked to colorectal cancer in ecological studies (Howell, 1975; Rose et al, 1986) and in a number of analytical epidemiological studies (Willett, 1989; Willett et al, 1990). The latter suggest also that a diet low in vegetables and fruit is associated with increased colorectal cancer risk (Willett, 1989). Various dietary constituents have been proposed to explain the above associations. These include saturated fat (Willett et al, 1990), total calories (Lyon et al, 1987), polycyclic or heterocyclic amines (Sinha et al, 1994), dietary fibre (Trock et al, 1990), antioxidant vitamins (Byers and Perry, 1992), calcium and vitamin D (Bostick, 1993), but the evidence supporting these hypotheses is incon-clusive.

Various naturally occurring constituents in vegetables and fruits have been studied for their chemopreventive potential against colorectal cancer. Among those, folate has recently been the subject of much research interest. Folate is essential for one-carbon transfer reactions in the normal synthesis and metabolism of amino acids, purines, pyrimidines and lipids. It is also a cofactor in the production of S-adenosylmethionine, the primary methyl donor in the body (Cooper, 1983). The S-adenosylmethionine-dependent methylation of specific DNA cytosine bases to form 5-methylcytosine has been shown to block gene expression, as well as the abnormal expression of proto-oncogenes (Nyce et al, 1983; Hoffman, 1984). Increases in the levels of messenger RNA for the proto-oncogenes c-*fos*, c-*HA-ras*, and c-*myc* have been found to be correlated with the loss of methylation in the liver of rats on a low methyl diet (Wainfan and Poirier, 1992). Hypomethylation has been observed in selected genes (Feinberg and Vogelstein, 1983; Goelz et al, 1985) as well as in total genomic contents (Feinberg et al, 1988) in human colonic adenocarcinomas and premalignant adenoma. In rats, folate deficiency-induced DNA strand breaks and hypomethylation within the *p*53 tumor suppressor gene (Kim et al, 1997). A moderate folate deficiency also enhanced the incidence of colonic dysplasia and neoplasia in rats treated with dimethylhydrazine (Cravo et al, 1992), which was preceded by alterations in the levels of S-adenosylmethionine and in the activity of specific methyl transferase enzymes (Halline et al, 1988). Folate supplementation led to a progressive reduction in the evolution of macroscopic colonic neoplasia from microscopic foci in a rat model (Kim et al, 1996).

In epidemiological studies, a lower risk of colorectal adenoma has been observed for individuals with a high folate intake (Benito et al, 1993; Giovannucci et al, 1993; Tseng et al, 1996; Baron et al, 1998) or high blood folate levels (Bird et al, 1995; Paspatis et al, 1995). Prospective (Giovannucci et al, 1995; Glynn et al, 1996) and case–control studies of colorectal cancer (Freudenheim et al, 1991; Benito et al, 1991; Ferraroni et al, 1994) have also shown

*Received 22 July 1998*
*Revised 18 September 1998*
*Accepted 8 October 1998*

*Correspondence to:* I Kato some inverse association with folate intake. In addition, red cell folate was inversely associated with the risk of developing colonic dysplasia or cancer among patients with ulcerative colitis (Lashner, 1993). Another prospective study found that lower plasma folate levels were associated with the risk of colorectal cancer among subjects who had a homozygous mutation in the 5, 10-methylenetetrahydrofolate reductase (MTHFR) gene, which is critical for folate metabolism (Ma et al, 1997). Some of the earlier studies have also suggested that high alcohol or low protein/methionine intake may modify the effect of folate (Giovannucci et al, 1993, 1995; Glynn et al, 1996).

Overall evidence in support of a role for folate in the aetiology of colorectal cancer is still limited and few studies have utilized biological markers, such as blood folate or homocysteine levels.

### MATERIALS AND METHODS

### Study population

Study subjects were women who had volunteered to participate in the New York University Women's Health Study. Details concerning cohort eligibility, procedures of data collection, follow-up and dietary assessment have been published previously (Toniolo et al, 1991, 1994, 1995; Kato et al, 1997). Briefly, the original study population consisted of 15 785 women enrolled in the study between 1985 and 1991 in New York City ($n$ = 14 275) or at a collaborating institution in Florida ($n$ = 1510). Women who in the preceding 6 months had neither used hormonal medications nor been pregnant were eligible for the study. At enrolment, written informed consent was obtained; basic demographic, medical, anthropometric, reproductive and dietary data were collected through self-administered questionnaires; and 30 ml of nonfasting peripheral venous blood was drawn.

After the initial examination, the cohort was followed-up through mailed questionnaires in order to identify incident cases of cancer diagnosed prior to 1995 and to update some important epidemiological risk factors. Telephone interviews were conducted if subjects failed to respond to the mailed questionnaire. Medical records were obtained from hospitals and reviewed to confirm pathological diagnoses for self-reported cancer. Record linkage with state cancer registries in New York, New Jersey and Connecticut and with the National Death Index supplemented the active follow-up. The results of capture–recapture analysis (Hook and Regal, 1995) based on the cases among New York residents indicated 94% completeness of follow-up.

### Nested case–control study

A total of 105 cases of colorectal cancer diagnosed before the end of 1994 was identified and included in a case–control study nested within the cohort. This number was very close to the expected number of cases based on population-based registry data, which was 100. The subsite distribution of the 105 cancers was as follows: 25 right colon (caecum, appendix, ascending colon and hepatic flexure); 8 transverse colon; 38 left colon (splenic flexure, descending colon and sigmoid colon); 11 rectosigmoid junction; 17 rectum and six unspecified colon. The criteria for control selection were identical to those developed for a breast cancer case–control study in the same cohort and have been described (Toniolo et al, 1995; Kato et al, 1997). Briefly up to five controls per case, matched by age, menopausal status at enrolment, date of enrolment and dates of subsequent blood donations, were randomly selected from among the cohort members who were alive and free of colorectal cancer at the time of case diagnosis. Premenopausal subjects were also matched by day and phase of the menstrual cycle at enrolment. A total of 523 individually matched controls were selected. Cases and controls were contacted by telephone by an interviewer (who was unaware of their case–control status) to obtain information on colorectal cancer risk factors in more detail than at the cohort baseline.

### Laboratory methods

After blood samples were drawn and centrifuged, the serum was partitioned into 1-ml aliquots and immediately stored at –80°C until biochemical assays were performed. Aliquots not previously thawed were used for this study.

Serum folate was measured using an automated clinical immunoassay analyser, the Technicon immuno 1® System from Bayer Corp (Letellier et al, 1996). Serum homocysteine was quantified by high performance liquid chromatography (HPLC) described by Vester and Rasmussen (1991). To ensure comparable laboratory measurements within matched sets, serum specimens from a given matched set were always assayed in the same batch, with the laboratory technician blind as to which were from cases and which from controls. The intra-assay coefficients of variation for the standards at the level nearest to the mean concentrations of folate and homocysteine in our population were 2.8% and 2.6%, respectively.

### Folate intake

Dietary folate intake was estimated by using a self-administered, semi-quantitative diet questionnaire consisting of 70 items of typical American foods, adapted from a questionnaire developed and validated at the National Cancer Institute (Block et al, 1986). Details concerning dietary assessment have been described elsewhere (Kato et al, 1997). Study subjects were also asked to provide information on their use of vitamin/mineral supplements, including the number of pills consumed, the frequency of use, and brand names. Dosage was asked only for individual preparations of vitamins A, C and E. Daily intake of folate supplements was calculated based on the chemical formulation of the brands, the number of pills consumed and the frequency of intake. For subjects who could not specify the name of their brand, the mean dose of all other brands used by the study subjects was applied.

### Statistical analysis

The variables which were tested in this study as predictors of the risk of colorectal cancer are serum folate, serum homocysteine and total folate intake (diet + supplements). In order to reduce departures from the normal distribution, the measurements of serum folate, homocysteine and total folate intake were log-transformed. In univariate analyses, the measurements for individual cases were compared with the mean levels for their matched controls using a paired $t$-test. Odds ratios (OR) for colorectal cancer by quartiles of these measurements and 95% confidence intervals (CI) were computed from conditional logistic regression models (Breslow and Day, 1990). Cut-off points for quartiles were obtained from the combined distribution of cases and controls included in particular analyses. Tests for linear trend in the logit of risk were calculated from conditional logistic regression models using

© Cancer Research Campaign 1999

**Table 1** Characteristics of study subjects

| Characteristics | Mean ± SD or percent | |
|---|---|---|
| | Cases | Controls |
| No. of subjects | 105 | 523 |
| Age at study entry (years) | 61.6 ± 7.5 | 61.6 ± 7.4 |
| Age at diagnosis (years) | 66.2 ± 8.6 | – |
| White | 85.4% | 86.2% |
| Jewish | 48.3% | 51.0% |
| Graduate school | 15.7% | 21.8% |
| Current smoker | 7.9% | 14.4% |
| Quetelet index[a] | 26.1 ± 4.9 | 25.6 ± 4.6 |
| Alcohol intake (drinks/week) | 1.89 ± 4.7 | 1.89 ± 6.4 |
| Calorie intake (kcal/day) | 1500 ± 581 | 1490 ± 651 |
| Vitamin/mineral supplement use | 60.2% | 71.3% |
| Regular aspirin use | 6.3% | 14.4% |

[a]Weight/height (kg $m^{-2}$).

ordered quartile categories. The analyses were repeated for the following subsites: proximal colon (right + transverse colon), distal colon (left colon + rectosigmoid junction) and rectum.

Several potential confounders other than the matching variables were first screened individually in the model. These included demographic variables (education, race, religion), physical activity levels at two time points (5 years before diagnosis and in their early 30s), history of regular aspirin use, family history of colorectal cancer, alcohol consumption (total, beer, wine and liquor), cigarette smoking, history of occult blood testing, total calorie intake, intakes of calorie-adjusted macronutrients (carbohydrate, protein and fat) and of dietary fibre, total intake of vitamins A, C and E, height and Quetelet index (weight in kilograms divided by height in $m^2$). Variables showing associations with the risk of colorectal cancer at $P < 0.10$ were further tested in forward stepwise-conditional regression models. The final model consisted of those variables showing a significant association with the risk of colorectal cancer at $P < 0.05$ level in the stepwise multiple regression model, i.e. beer intake (0, <3 or ≥ 3 cans per week), sport activities in their early 30s (0, <3 or ≥ 3 h per week), history of occult blood testing, and family history of colorectal cancer among first-degree relatives. Reported adjusted odds ratios were computed using this model.

### RESULTS

Selected characteristics of the cases and controls are presented in Table 1. The cases and controls were similar in age, race and religion. They had similar levels of total calorie and alcohol consumption and similar body mass induces. Controls tended to be more educated, and more likely to be taking vitamin/mineral supplements and aspirin.

In univariate analyses (Table 2), the cases had significantly lower serum folate and higher serum homocysteine levels than controls. The difference was more pronounced for folate ($P < 0.001$) than homocysteine ($P = 0.044$). When analysed by subsite, the differences were greatest for the distal colon, followed by the proximal colon and rectum. To assess whether the presence of preclinical cancer may have affected the levels of serum markers, we examined whether serum folate and homocysteine concentrations among the cases were associated with the length of time between the drawing of blood and diagnosis. However, there were no apparent increasing or decreasing trends in the concentration of either folate or homocysteine as the date when the blood sample was obtained approached the date of diagnosis (data not shown).

The odds ratios for colorectal cancer in uni- and multivariate analyses are shown in Table 3. The adjustment for potential confounders made no substantial change in the ORs associated with serum folate and homocysteine. Among those in the highest quartile of serum folate (≥ 31.1 nmol $l^{-1}$), the risk of developing colorectal cancer was about half that of those in the lowest quartile (≤12.2 nmol $l^{-1}$). The risk of colorectal cancer decreased with increasing serum folate levels ($P$-value for trend = 0.04). When the seven cases who were diagnosed within 1 year of their blood sample were excluded, the odds ratio did not change (0.50, 95% CI: 0.26–0.96, $P$-value for trend = 0.04). For homocysteine, subjects in the highest quartile (> 12.2 μmol $l^{-1}$) had a 70% increase in risk of colorectal cancer compared with those in the lowest quartile (≤ 7.9 μmol $l^{-1}$) ($P = 0.09$). The associations were strongest for distal colon, followed by proximal colon and rectum (data not shown). The inverse association between serum folate and colorectal cancer risk was observed for both vitamin/mineral supplement users and nonusers. The odds ratios for the highest compared to the lowest quartile of serum folate levels were 0.54 (95% CI: 0.22–1.35) among supplement users (62 sets) and 0.18 (95% CI: 0.02–1.73) among nonusers (31 sets), using separate quartile levels for users and nonusers.

**Table 2** Differences in serum levels of folate and homocysteine and total intake of folate between colorectal cases and matched controls

| Variables | All sites (105 sets) | Proximal colon (33 sets) | Distal colon (49 sets) | Rectum (17 sets) |
|---|---|---|---|---|
| Serum folate (nmol $l^{-1}$) | | | | |
| Geometric mean of cases | 17.08 | 17.58 | 15.96 | 18.43 |
| Mean difference (case–controls) | –5.36 | –4.31 | –7.59 | –2.10 |
| *P*-value | <0.001 | 0.203 | 0.001 | 0.662 |
| Serum homocysteine (μmol $l^{-1}$) | | | | |
| Geometric mean of cases | 10.35 | 11.30 | 10.02 | 9.62 |
| Mean difference (case–controls) | 0.67 | 0.72 | 0.89 | 0.15 |
| *P*-value | 0.044 | 0.352 | 0.022 | 0.805 |
| Total folate intake (μg $day^{-1}$) | | | | |
| Geometric mean of cases | 354.2 | 404.6 | 309.8 | 385.3 |
| Mean difference (case–controls) | –21.9 | 51.8 | –70.5 | –24.6 |
| *P*-value | 0.476 | 0.413 | 0.076 | 0.762 |

*© Cancer Research Campaign 1999*

**Table 3** Univariate and multivariate odds ratios for colorectal cancer according to quartile levels of serum folate and homocysteine and total folate intake

| | Univariate | | Multivariate[a] | |
|---|---|---|---|---|
| Variables | OR | 95% CI | OR | 95% CI |
| Serum folate (nmol $l^{-1}$) | | | | |
| ≤ 12.23 | 1.00 | — | 1.00 | — |
| 12.24–19.25 | 0.66 | 0.38–1.17 | 0.67 | 0.37–1.23 |
| 19.26–31.03 | 0.60 | 0.34–1.06 | 0.66 | 0.36–1.21 |
| ≥31.04 | 0.46 | 0.25–0.85 | 0.52 | 0.27–0.97 |
| *P* for trend | 0.01 | | 0.04 | |
| Serum homocysteine (μmol $l^{-1}$) | | | | |
| ≤ 7.9 | 1.00 | — | 1.00 | — |
| 7.91–9.90 | 1.04 | 0.53–2.07 | 1.06 | 0.52–2.17 |
| 9.91–12.2 | 1.66 | 0.84–3.28 | 1.51 | 0.74–3.08 |
| ≥ 12.21 | 1.73 | 0.86–3.45 | 1.72 | 0.83–3.57 |
| *P* for trend | 0.07 | | 0.09 | |
| Total folate intake (μg $day^{-1}$) | | | | |
| ≤224 | 1.00 | | 1.00 | |
| 225–413 | 0.78 | 0.42–1.44 | 0.92 | 0.48–1.74 |
| 414–625 | 0.70 | 0.38–1.29 | 0.87 | 0.45–1.70 |
| ≥ 626 | 0.67 | 0.36–1.23 | 0.88 | 0.46–1.69 |
| *P* for trend | 0.17 | | 0.67 | |

[a]Adjusted for family history of colorectal cancer, beer intake, prior occult blood testing and number of hours spent in sport activities in their early 30s.

In analyses of folate intake from diet and supplements, the overall differences between cases and controls were small, but the cases with distal colon cancer tended to consume less folate than their matched controls ($P = 0.08$) (Table 2). Correspondingly, the univariate OR for the highest quartile of total folate intake (≥ 626 mg $day^{-1}$) compared with the lowest (≤ 224 mg $day^{-1}$) was 0.32 (95% CI: 0.11–0.93, *P*-value for trend = 0.01) for distal colon cancer. Adjusting for confounders weakened the OR to 0.43 (95% CI: 0.14–1.39, *P*-value for trend = 0.18) (data not shown).

In our study population, supplemental folate represented 39% of the total folate intake. We therefore examined whether the use of vitamin/mineral supplements was associated with colorectal cancer risk. The OR associated with any vitamin/mineral supplement use was 0.62 (95% CI: 0.40–0.96) in univariate and 0.67 (95% CI: 0.42–1.06) in multivariate analyses.

There was no statistical interaction between alcohol consumption and serum folate levels. However, participants who were in the lower half of serum folate and the upper half of total alcohol consumption had a risk of developing colorectal cancer almost twice as high as those who were in the upper half of serum folate and the lower half of total alcohol consumption (OR = 1.99, 95% CI: 0.92–4.29). No such increase in risk was observed for the combination of low serum folate and low protein intake.

The Spearman rank correlation coefficients among the three folate indices were 0.52 between total folate intake and serum folate, –0.36 between serum folate and homocysteine and – 0.26 between total folate intake and serum homocysteine.

### DISCUSSION

Despite uncertainties regarding the underlying biological mechanisms, several lines of laboratory evidence suggest that folate may be protective against colorectal cancer. To our knowledge, the present epidemiological study is the first to assess prospectively the relationship between biological markers for folate status and colorectal cancer risk among women. The blood specimens used for this analysis were taken between 2.4 months and 9.1 years before the subjects were diagnosed with colorectal cancer. The study found a progressive decrease in risk of colorectal cancer with increasing serum folate levels and with decreasing homo cysteine levels.

Two other prospective studies have assessed the relationship between serum/plasma folate levels and colorectal cancer risk in selected male populations, Finnish smokers and US physicians (Glynn et al, 1996; Ma et al, 1997). While the study in Finnish smokers found no association (Glynn et al, 1996), the US physicians' study found a marginally significant increased risk in men with a severe depletion in plasma folate levels (Ma et al, 1997). Subjects in both studies had taken some intervention materials, such as antioxidant vitamins or aspirin, so that the effect of folate may have been confounded by such treatments. There have been some small earlier cross-sectional studies of subjects undergoing colonoscopic examinations which looked at biological markers for folate status. Two such studies found an inverse association of colorectal adenoma or dysplasia with red cell folate levels for both genders combined (Lashner, 1993; Paspatis et al, 1995), while another showed an inverse association with plasma folate in men, but not in women (Bird et al, 1995). These studies, however, cannot distinguish between the causes and consequences of a disease. Other such studies based on either total or dietary folate intake have yielded mixed results (Benito et al, 1991, 1993; Freudenheim et al, 1991; Giovannucci et al, 1993; Ferraroni et al, 1994; Tseng et al, 1996; Baron et al, 1998). Overall, the decrease in risk of colorectal cancer for subjects in the highest quartile or quintile levels of the folate measurements ranged from 75% to 35%. These reductions in risk are comparable with the odds ratio of 0.52 observed in the present study.

Previous studies have also suggested interactions of folate intake with alcohol and with protein/methionine. Ethanol and its metabolites can lower blood folate levels through several different mechanisms, such as promoting catabolism, inhibiting absorption and increasing excretion (Eichner and Hillman, 1971; Romero et

 © Cancer Research Campaign 1999

al, 1981; Shaw et al, 1989). Methionine, an amino acid derived from protein, and folate are both essential for the production of S-adenosylmethionine (Cooper et al, 1983). In the two recent prospective studies, a significant increase in risk of colorectal cancer associated with lower folate intake was only observed in the combination with higher alcohol and lower methionine/protein intake (Giovannucci et al, 1995; Glynn et al, 1996). In our study, despite the lack of association with total alcohol intake, there was a marginal indication that the combination of higher alcohol and lower serum folate levels may increase the risk of colorectal cancer in women. However, it should be noted that the mean alcohol intake in the New Women's Health Study cohort was relatively low compared with that in other cohorts, so that it may not be the most appropriate for a study of the interaction with alcohol.

Three measurements were used to assess folate status in the present study and while an association with colorectal cancer risk was found with the two serum markers, there was almost no association with total folate intake. These discrepancies may be explained in several different ways: (1) the dose from internal exposure (blood folate) is not perfectly correlated with that from external exposure (intake) because of differences in absorption and metabolism. The Spearman rank correlation between total intake and serum levels in our study subjects was 0.52. Some recent studies reported that certain mutations in the gene of a folate-related enzyme, MTHFR, which led to reduced blood folate levels, were associated with the risk of colorectal cancer (Chen et al, 1996; Ma et al, 1997). (2) Measurement errors in total folate intake may have reduced differences between cases and controls. Although our dietary questionnaire consisting of 70 food items was adapted from a questionnaire designed to capture over 90% of 17 major nutrient intakes (Block et al, 1986), it was less comprehensive than those being used more recently (Benito et al, 1991, 1993; Giovannucci et al, 1993, 1995; Bird et al, 1995; Glynn et al, 1996; Tseng et al, 1996; Baron et al, 1998). However, the estimated dietary and total folate intakes were comparable with, or higher than, those based on more comprehensive questionnaires (Benito et al, 1991, 1993; Giovannucci et al, 1993, 1995; Bird et al, 1995; Glynn et al, 1996; Tseng et al, 1996; Baron et al, 1998). In a reproducibility study of our dietary questionnaire, in which it was administered to 267 cohort members after an interval of 2–3 months, the Spearman rank correlation for dietary folate was 0.66.

Because serum folate tends to reflect the short-term balance of folate, red-cell folate (which is an indicator of tissue levels) may be a more appropriate index of chronic folate deficiency. To compensate for the lack of red-cell specimens in our study, we measured serum homocysteine, which has been proposed as a sensitive indicator of functional folate deficiency that is distinguishable from low serum folate concentrations following short-term decreases in dietary intake (Kang et al, 1987; Stabler et al, 1988; Ubbink et al, 1993; Jacob et al, 1994; O'Keefe et al, 1995). Our data show that the variability of serum homocysteine in the population was much narrower than that of folate, suggesting that homocysteine levels are more tightly regulated by metabolism. Recent studies show that factors other than folate, such as sex steroids, also affect serum homocysteine levels (Giltay et al, 1998). Thus, homocysteine seems to be a less sensitive indicator of mean folate intake than is direct measurement of serum folate.

Several limitations in the present study arise from the fact that the original study was designed for breast cancer. For example, because of the relatively small number of colorectal cancer cases, our study had limited statistical power for analyses by subsite of risk factors that have been reported by other authors Broeders et al, 1996; Lund, 1996; (Thune and Wurzelmann et al, 1996). Secondly, epidemiological variables collected at baseline were mostly potential risk factors for breast cancer. Those specific to colorectal cancer were collected retrospectively on a case–control basis by telephone interview and biases in the measurement of confounding factors, which are typically seen in case–control studies, may have affected risk estimates. Third, because the follow-up was relatively short (average 4.7 years), some cases may have had preclinical cancer when their blood sample was taken and this may have influenced serum folate and homocysteine levels. However, the exclusion of cases diagnosed within 1 year of blood donation did not alter the results. Finally, as our study subjects were participants in breast cancer screening, they were likely to be more health conscious (Kato et al, 1986, 1987) and more homogeneous than the general population, as was subsequently shown by the high prevalence of vitamin/mineral supplement use. Consequently, the number of subjects whose serum levels were indicative of folate deficiency (< 6.8 nmol $l^{-1}$) was too small (4.5%) for analysis of the effect of very low levels. Also, because the majority of our study subjects was taking multi-vitamin pills, it is possible that serum folate serves as a marker for other vitamins in the blood. For the above reasons and because our cohort is self-selected and consists of a middle-class and largely Caucasian population, caution needs to be exercised in generalizing the results.

In summary, the results of the present study support the hypothesis that folate may be protective against colorectal cancer. However, due to the limitations of observational epidemiological studies, the potentially protective effects of folate against colorectal cancer need to be addressed in clinical trials.

### ACKNOWLEDGEMENTS

This study was supported by Research Grants CA69201, CA34588 and CA16087 from the National Cancer Institute and ES00260 from the National Institute of Environmental Health Sciences. The authors thank Ms Nicole Heitler, Lynne Quinones, Joan Szymczak and Mr Ellery Aziel for their assistance.

### REFERENCES

American Cancer Society (1997) *Cancer Facts and Figures*

Baron JA, Sandler RS, Haile RW, Mandel JS, Mott LA and Greenberg ER (1998) Folate intake, alcohol, cigarette smoking of colorectal adenomas. *J Natl Cancer Inst* **90**: 57–62

Benito E, Cabeza E, Moreno V, Obrador A and Bosch FX (1993) Diet and colorectal adenomas: a case-control study in Majorca. *Int J Cancer* **55**: 213–219

Benito E, Stiggelbout A, Bosch FX, Obrador A, Kaldor J, Mulet M and Munoz M (1991) Nutritional factors in colorectal cancer risk: a case-control study in Majorca. *Int J Cancer* **49**: 161–167

Bird CL, Swendseid ME, Witte JS, Shikany JM, Hunt IF, Frankl HD, Lee ER, Longnecker MP and Haile RW (1995) Red cell and plasma folate, folate consumption, and risk of colorectal adenomatous polyps. *Cancer Epi Bio Prev* **4**: 709–714

Block G, Hartman AM, Dresser CM, Carroll MD, Gannon J and Gardner L (1986) A data-based approach to diet questionnaire design and testing. *Am J Epidemiol* **124**: 453–469

Bostick RM, Potter JD, Sellers TA, McKenzie DR, Kushi LH and Folsom AR (1993) Relation of calcium, vitamin D, and dairy food intake to incidence of colon cancer among older women: The Iowa Women's Health Study. *Am J Epidemiol* **137**: 1302–1317

Breslow NE and Day NE (1990) Conditional logistic regression for matched sets. In *Statistical Methods in Cancer Research. Vol 1. The Analysis of Case-Control Studies*, pp 248–279. International Agency for Research on Cancer: Lyon

© Cancer Research Campaign 1999 

Broeders MJM, Lambe M, Baron JA and Leon DA (1996) History of childbearing and colorectal cancer risk in women aged less than 60: an analysis of Swedish routine registry data 1960–1984. *Int J Cancer* **66**: 170–175

Byers T and Perry G (1992) Dietary carotenes, vitamin C, and vitamin E as protective antioxidants in human cancers. *Ann Rev Nutr* **12**: 139–159

Chen J, Giovannucci E, Kelsey K, Rimm EB, Stampfer MJ, Colditz GA, Spiegelman D, Willett WC and Hunter DJ (1996) A methylenetetrahydrofolate reductase polymorphism and the risk of colorectal cancer. *Cancer Res* **56**: 4862–4864

Cooper AJ (1983) Biochemistry of sulfur-containing amino acids. *Annu Rev Biochem* **52**: 187–222

Cravo ML, Mason JB, Dayal Y, Hutchinson M, Smith D, Selhub J and Rosenberg IH (1992) Folate deficiency enhances the development of colonic neoplasia in dimethylhydrazine-treated rats. *Cancer Res* **52**: 5002–5006

Doll R and Peto R (1981) The causes of cancer: quantitative estimates of avoidable risks of cancer in the United States today. *J Natl Cancer Inst* **66**: 1191–1308

Eichner ER and Hillman RS (1971) The evolution of anemia in alcoholic patients. *Am J Med* **50**: 218–232

Feinberg AP, Gehrke CW, Kuo KC and Ehrlich M (1988) Reduced genomic 5-methylcytosine content in human colonic neoplasia. *Cancer Res* **48**: 1159–1161

Feinberg AP and Vogelstein B (1983) Hypomethylation distinguishes genes of some human cancers from their normal counterparts. *Nature* **301**: 89–92

Ferraroni M, La Vecchia C, D'Avanzo B, Negri E, Franceschi S and Decarli A (1994) Selected micronutrient intake and the risk of colorectal cancer. *Br J Cancer* **70**: 1150–1155

Freudenheim JL, Graham S, Marshall JR, Haughey BP, Cholewinski S and Wilkinson G (1991) Folate intake and carcinogenesis of the colon and rectum. *Int J Epidemiol* **20**: 368–374

Giltay EJ, Hoogeveen EK, Elbers JMH, Gooren LJG, Asscheman H and Stehouwer CDA (1998) Effects of sex steroids on plasma total homocysteine levels: a study in transsexual males and females. *J Clin Endocrinol Metab* **83**: 550–553

Giovannucci E, Rimm EB, Ascherio A, Stampfer MJ, Colditz GA and Willett WC (1995) Alcohol, low-methionine-low-folate diets, and risk of colon cancer in men. *J Natl Cancer Inst* **87**: 265–273

Giovannucci E, Stampfer MJ, Colditz GA, Rimm EB, Trichopoulos D, Rosner BA, Spizer FE and Willett WC (1993) Folate, methionine, alcohol intake and risk of colorectal adenoma. *J Natl Cancer Inst* **85**: 875–884

Glynn SA, Albanes D, Pietinenn P, Brown CC, Rautalahti M, Tangrea JA, Gunter EW, Barreett MJ, Virtamo J and Taylor PR (1996) Colorectal cancer and folate status: a nested case-control study among male smokers. *Cancer Epi Bio Prev* **5**: 487–494

Goelz SE, Vogelstein B, Hamilton SR and Feinberg AP (1985) Hypomethylation of DNA from benign and malignant human colon neoplasms. *Science* **228**: 187–190

Halline AG, Dudeja PK and Brasitus TA (1988) 1,2-dimethylhydrazine-induced premalignant alterations in the S-adenosylmethionone/S-adenosylhomocysteine ratio and membrane lipid lateral diffusion of the rat distal colon. *Biochim Biophys Acta* **944**: 101–107

Hoffman RM (1984) Altered methionine metabolism, DNA methylation and oncogene expression in carcinogenesis. *Biochim Biophys Acta* **738**: 49–87

Hook EB and Regal RR (1995) Capture-recapture methods in epidemiology: methods and limitations. *Epidemiol Rev* **17**: 243–264

Howell MA (1975 Diet as an etiological factor in the development of cancers of the colon and rectum. *J Chron Dis* **28**: 67–80

Jacob RA, Wu M-M, Henning SM and Swendseid ME (1994) Homocysteine increases as folate decreases in plasma of healthy men during short-term dietary folate and methyl group restriction. *J Nutr* **124**: 1072–1080

Kang SS, Wong PWK and Norusis MR (1987) Hyperhomocysteinemia due to folate deficiency. *Metabolism* **36**: 458–462

Kato I, Akhmedkhanov A, Koenig K, Toniolo P, Shore RE and Riboli E (1997) Prospective study of diet and female colorectal cancer. *Nutr Cancer* **28**: 276–281

Kato I, Tominaga S and Matsuoka I (1987) Characteristics of participants of uterine cancer screening test. *Jpn J Public Health* **34**: 748–754

Kato I, Tominaga S and Naruhashi H (1986) Characteristics of the participants of stomach cancer screening test. *Jpn J Public Health* **33**: 749–753

Kim Y-I, Pogribny IP, Basnakian AG, Miller JW, Selhub J, James SJ and Mason JB (1997) Folate deficiency in rats induces DNA strand breaks and hypomethylation within the p53 tumor suppressor gene. *Am J Clin Nutr* **65**: 46–52

Kim Y-I, Salomon RN, Graeme-Cook F, Choi S-W, Smith DE, Dallal GE and Mason JB (1996) Dietary folate protects against the development macroscopic colic neoplasia in a dose-response manner in rats. *Gut* **39**: 732–740

Lashner BA (1993) Red blood cell folate is associated with the development of dysplasia and cancer in ulcerative colitis. *J Cancer Res Clin Oncol* **119**: 549–554

Letellier M, Levesque A, Daigle F and Grant A (1996) Performance evaluation of automated immunoassays on the Technicon Immuno 1 system. *Clin Chem* **42**: 1695–1701

Lyon JL, Mahoney AW, West DW, Gardner JW, Smith KR, Sorenson AW and Stanish W (1987) Energy intake: its relationship to colon cancer risk. *J Natl Cancer Inst* **78**: 853–861

Ma J, Stampfer MJ, Giovannucci E, Artigas C, Hunter DJ, Fuchs C, Willett WC, Selhub J, Hennekens CH and Rozen R (1997) Methylenetetrahydrofolate reductase polymorphism, dietary interactions, and risk of colorectal cancer. *Cancer Res* **57**: 1098–1102

Nyce J, Weinhouse S and Magee PN (1983) 5-methylcytosine depletion during tumor development: an extension of the miscoding concept. *Br J Cancer* **48**: 463–475

O'Keefe CA, Bailey LB, Thomas EA, Hofler SA, Davis BA, Cerda JJ and Gregory JF (1995) Controlled dietary folate affects folate status in nonpregnant women. *J Nutr* **125**: 2717–2725

Paspatis GA, Kalafatis E, Oros L, Xourgias V, Koutsioumpa P and Karamanolis D (1995). Folate status and adenomatous colonic polyps: a colonoscopically controlled study. *Dis Colon Rectum* **38**: 64–68

Romero JJ, Tamura T and Halsted CH (1981) Intestinal absorption of [$^3$H]folic acid in the chronic alcoholic monkeys. *Gastroenterology* **80**: 99–102

Rose DP, Boyar AP and Wynder EL (1986) International comparisons of mortality rates for cancer of the breast, ovary, prostate, and colon, and per capita food consumption. *Cancer* **58**: 2363–2371

Shaw S, Jayatilleke E, Herbert V and Colman N (1989) Cleavage of folates during ethanol metabolism. Role of acetaldehyde/xanthine oxidase-generated superoxide. *Biochem J* **257**: 277–280

Sinha R, Rothman N, Brown ED, Mark SD, Hoover RN, Caporaso NE, Levander OA, Knize MG, Lang NP and Kadlubar FF (1994) Pan-fried meat containing high levels of heterocyclic aromatic amines but low levels of polycyclic aromatic hydrocarbons induces cytochrome P4501A2 activity in humans. *Cancer Res* **54**: 6154–6159

Stabler SP, Marcell PD, Podell ER, Allen RH, Savage DG and Lindenbaum J (1988) Elevation of total homocysteine in the serum of patients with cobalamin or folate deficiency detected by capillary gas chromatography-mass spectrometry. *Clin Invest* **81**: 466–474

Thune I and Lund E (1996) Physical activity and risk of colorectal cancer in men and women. *Br J Cancer* **73**: 1134–1140

Toniolo PG, Levitz M, Zeleniuch-Jacquotte A, Banerjee S, Koenig KL, Shore RE, Strax P and Pasternack BS (1995) A prospective study of endogenous estrogens and breast cancer in postmenopausal women. *J Natl Cancer Inst* **87**: 190–197

Toniolo PG, Pasternack BS, Shore RE, Sonnenschein E, Koenig KL, Rosenberg C, Strax P and Strax S (1991) Endogenous hormones and breast cancer: a prospective study. *Breast Cancer Res Treat* **18**: S23–S26

Toniolo P, Riboli E, Shore RE and Pasternack BS (1994) Consumption of meat, animal products, protein, and fat and risk of breast cancer: a prospective cohort study in New York. *Epidemiology* **5**: 391–397

Trock B, Lanza E and Greenwald P (1990) Dietary fiber, vegetables, and colon cancer: critical review and meta-analysis of the epidemiologic evidence. *J Natl Cancer Inst* **82**: 650–661

Tseng M, Murray SC, Kupper LL and Sandler RS (1996) Micronutrients and the risk of colorectal cancer. *Am J Epidemiol* **144**: 1005–1014

Ubbink JB, Vermaak WJH, van der Merwe A, Becker PJ (1993) Vitamin B-12, vitamin B-6, and folate nutritional status in men with hyperhomocysteinemia. *Am J Clin Nutr* **57**: 47–53

Vester B and Rasmussen K (1991) High performance liquid chromatography method for rapid and accurate determination of homocysteine in plasma and serum. *Eur J Clin Chem Clin Biochem* **29**: 549–554

Wainfan E and Poirier LA (1992) Methyl groups in carcinogenesis: effects of DNA methylation and gene expression. *Cancer Res* **52**: 2071s–2077s

Willett W (1989) The search for the causes of breast and colon cancer. *Nature* **338**: 389–394

Willett W, Stampfer MJ, Colditz GA, Rosner BA and Spizer FE (1990) Relation of meat, fat and fiber intake to the risk of colon cancer in a prospective study among women. *N Engl J Med* **323**: 1664–1672

Wurzelmann JL, Silver A, Schreinemachers DM, Sandler RS and Everson RB (1996) Iron intake and the risk of colorectal cancer. *J Cancer Epi Bio Prev* **5**: 503–507

 © Cancer Research Campaign 1999
